# Shared Mental Models of Provider Roles in Cancer Survivorship Care

**DOI:** 10.6004/jadpro.2015.6.4.4

**Published:** 2015-07-01

**Authors:** Megan Hebdon, Olivia Fahnestock, Sara McComb

**Affiliations:** Purdue University School of Nursing, West Lafayette, Indiana; OHL Global Supply Chain Management and Logistics Solutions, Nashville, Tennessee; Purdue University School of Industrial Engineering, West Lafayette, Indiana

**Shared Mental Models of Provider Roles in Cancer Survivorship Care**

A continuing education article for nurse practitioners, physician assistants, clinical nurse specialists, advanced degree nurses, oncology and hematology nurses, pharmacists, and physicians.

**Release date:** July 15, 2015

**Expiration date:** July 15, 2016

**Expected time to complete this activity as designed:** 0.50 hours

**Meniscus Educational Institute**

3131 Princeton Pike,

Building 1, Suite 205A

Lawrenceville, NJ 08648

Voice: 609-246-5000

Fax: 609-449-7969

E-mail: lrubin@meniscusedu.com

**Journal of the Advanced Practitioner in Oncology**

37 Main Street

Cold Spring Harbor, NY 11724

Voice: 631-692-0800

Fax: 631-692-0805

E-mail: claudine@harborsidepress.com

© *2015, Meniscus Educational Institute. All rights reserved.*

## Faculty

**Megan Hebdon, DNP, RN, NP-C,** Purdue University School of Nursing

**Olivia Fahnestock, BS,** OHL Global Supply Chain Management and Logistics Solutions

**Sara McComb, PhD,** Purdue University School of Nursing and School of Industrial Engineering

## Activity Rationale and Purpose

The purpose of this article is to provide an integrative review to analyze cancer survivor, primary care provider, and oncology provider views in survivorship care using the conceptual framework of shared mental models.

## Intended Audience

The activity’s target audience will consist of nurse practitioners, physician assistants, clinical nurse specialists, advanced degree nurses, oncology and hematology nurses, pharmacists, and physicians.

## Learning Objectives

After completing this educational activity, participants should be able to:

1. Define the concept of "shared mental models," and list the factors that comprise such models
2. Describe how shared mental models contribute to the provision of survivorship care in cancer patients
3. Outline how oncologists, primary care providers, and cancer survivors perceive the roles of oncologists and primary care providers in cancer survivorship care
4. Describe the key barriers to the provision of optimal survivorship care
5. List the potential advantages of a written survivorship care plan
6. Identify ways in which survivorship care can be improved at their own institution.

## Continuing Education

**Statement of Credit—Participants who successfully complete this activity (including the submission of the post-test and evaluation form) will receive a statement of credit.**

**Physicians.** The Meniscus Educational Institute is accredited by the Accreditation Council for Continuing Medical Education (ACCME) to provide continuing medical education for physicians.

The Meniscus Educational Institute designates this journal article for a maximum of 0.50 AMA PRA Category 1 Credits™. Physicians should claim only the credit commensurate with the extent of their participation in the activity.

**Nurses.** This activity for 0.50 contact hours is provided by the Meniscus Educational Institute.

The Meniscus Educational Institute is accredited as a provider of continuing nursing education by the American Nurses Credentialing Center’s Commission on Accreditation.

Provider approved by the California Board of Registered Nursing, Provider No. 13164, for 0.50 contact hours.

**Pharmacists.** The knowledge-based accredited education lectures are intended for pharmacists involved in the care of cancer patients. This educational activity is sponsored by the Meniscus Educational Institute.

The Meniscus Educational Institute is accredited by the Accreditation Council for Pharmacy Education (ACPE) as a provider of continuing pharmacy education. The ACPE Universal Activity Number assigned to this program, for 0.50 contact hours, is 0429-0000-15-009-H04-P.

## Financial Disclosures

All individuals in positions to control the content of this program (eg, planners, faculty, content reviewers) are expected to disclose all financial relationships with commercial interests that may have a direct bearing on the subject matter of this continuing education activity. Meniscus Educational Institute has identified and resolved all conflicts of interest in accordance with the MEI policies and procedures. Participants have the responsibility to assess the impact (if any) of the disclosed information on the educational value of the activity.

**Faculty**

**Megan Hebdon, DNP, RN, NP-C,** has nothing to disclose.

**Olivia Fahnestock, BS,** has nothing to disclose.

**Sara McComb, PhD,** has nothing to disclose.

**Lead Nurse Planner**

**Wendy J. Smith, ACNP, AOCN®,** has nothing to disclose.

**Planners**

**Jeannine Coronna** has nothing to disclose.

**Claudine Kiffer** has nothing to disclose.

**Terry Logan, CHCP,** has nothing to disclose.

**Pamela Hallquist Viale, RN, MS, CNS, ANP,** has nothing to disclose.

**Content Reviewers**

**Glenn Bingle, MD, PhD, FACP,** has nothing to disclose.

**Kate D. Jeffers, PharmD, BCOP,** has nothing to disclose.

**Jody Pelusi, PhD, FNP, AOCNP®,** has nothing to disclose.

**Wendy J. Smith, ACNP, AOCN®,** has nothing to disclose.

## Disclaimer

This activity has been designed to provide continuing education that is focused on specific objectives. In selecting educational activities, clinicians should pay special attention to the relevance of those objectives and the application to their particular needs. The intent of all Meniscus Educational Institute educational opportunities is to provide learning that will improve patient care. Clinicians are encouraged to reflect on this activity and its applicability to their own patient population.

The opinions expressed in this activity are those of the faculty and reviewers and do not represent an endorsement by Meniscus Educational Institute of any specific therapeutics or approaches to diagnosis or patient management.

## Product Disclosure

This educational activity may contain discussion of published as well as investigational uses of agents that are not approved by the US Food and Drug Administration. For additional information about approved uses, including approved indications, contraindications, and warnings, please refer to the prescribing information for each product.

## How to Earn Credit

To access the learning assessment and evaluation form online, visit www.meniscusce.com

**Statement of Credit:** Participants who successfully complete this activity (including scoring of a minimum of 70% on the learning assessment and complete and submit the evaluation form with an E-mail address) will be able to download a statement of credit.

## ABSTRACT

In 2012, the United States had an estimated 13.7 million cancer survivors, with a projected increase to 18 million by 2022. Little consensus exists regarding provider roles in cancer follow-up care. The purpose of this integrative review is to analyze cancer survivor, primary care provider, and oncology provider views of provider roles in survivorship care using the conceptual framework of shared mental models. Searches using CINAHL, PubMed, and the Cochrane Database identified 22 studies fitting inclusion criteria. Primary care providers and oncologists were identified as providers of wellness care and specialized cancer care for survivors, respectively. Care continuity and the need for psychosocial support were themes noted by all groups. Survivorship care plans were cited as a means to foster provider communication and coordination. Survivorship research and interventions should be guided by a teamwork approach, where provider and patient roles are understood and maintained through measures such as shared care and survivorship care plans. Clarity of provider roles within the health-care team and team communication has the potential to improve continuity of care for cancer survivors.

## ARTICLE

In 2012, the United States had an estimated 13.7 million individuals living with a history of cancer, and this number is projected to increase to 18 million by 2022 (American Cancer Society, 2014; de Moor et al., 2013). This volume of survivors places a great burden on the health-care system for management of their follow-up care needs. Cancer survivors have unique care issues that continue across the lifespan, including long-term and late effects related to cancer and its treatment, psychosocial concerns, risk of recurrence or secondary cancers, and employment and insurance issues (Hewitt, Greenfield, & Stovall, 2005). Some barriers to survivorship care provision include public policy, insurance constraints, limited clinical practice guidelines, poor care continuity and provider communication, and lack of consensus regarding provider roles in survivorship care (McCabe et al., 2013). In this review, we will address the consensus of provider roles in survivorship care.

## BACKGROUND

Recently, significant developments in research and public policy to support the care of cancer survivors have emerged. In 2004, the Centers for Disease Control and Prevention (CDC) and the Livestrong Foundation released *A National Action Plan for Cancer Survivorship: Advancing Public Health Strategies* (CDC, 2004; The Livestrong Foundation, 2010). This report established cancer survivorship as a public health issue and proposed measures to improve the quality of life of cancer survivors through prevention, chronic disease management, and resource access (CDC, 2004).

In 2005, the Institute of Medicine released *From Cancer Patient to Cancer Survivor: Lost in Transition*, which addressed the core components of cancer survivorship care including prevention, detection and surveillance for new or recurrent cancer, management of late effects, and care coordination between primary care and specialty providers (Hewitt et al., 2005). Survivorship care plans were introduced as a way to promote care continuity and provider communication. They have also gained support from both the Commission on Cancer and the Livestrong Foundation (American College of Surgeons, 2012; The Live-strong Foundation, 2010).

## SIGNIFICANCE

Resources to support evidence-based care and insurability of cancer survivors have been developed through the National Comprehensive Cancer Network (NCCN) and the Affordable Care Act. The NCCN recently released practice guidelines for specific survivorship concerns such as fatigue, anxiety and depression, exercise, pain, and sexual and cognitive dysfunction (NCCN, 2013). The Affordable Care Act has provisions that affect insurability and care for cancer survivors, including elimination of caps on annual and lifetime benefits, copays for preventive services, and preexisting condition clauses for new insurance plans; coordinated care through accountable care organizations and patient-centered medical homes; and limitations on allowable out-of-pocket spending amounts (McCabe et al., 2013).

Despite the growing resources for and awareness of cancer survivorship care, the lack of consensus regarding the role of care providers in cancer survivorship care is a barrier to developing evidence-based care interventions. A variety of survivorship care models have been implemented in clinical practice, with no pattern of research to substantiate the superiority of one specific model (Landier, 2009). Often, the models of care are specific to the needs of each setting, whether community or academic, and the mode of care delivery, organ specific vs. general care (Landier, 2009). When organizations are structuring survivorship care, it is essential to focus on some fundamental questions: How can organizations best utilize the roles, background, education, and expertise of care providers who are managing survivorship care? How can high-quality and low-cost survivorship care be provided given available resources? How can survivorship care be structured to align with the concept of patient-centered care (McCabe et al., 2013)?

## CONCEPTUAL FRAMEWORK

To facilitate an examination of the literature regarding provider roles in the provision of cancer survivorship care, we employed shared mental models as a conceptual framework. Shared mental models are coordinating mechanisms that facilitate teamwork (Salas, Sims, & Burke, 2005) and comprise content, similarity, accuracy, and dynamics (McComb & Simpson, 2013). Researchers have demonstrated that team members exhibiting similar mental models have better communication, actively engage in teamwork, and are more willing to work together again in the future (Salas et al., 2005).

**Figure 1:**
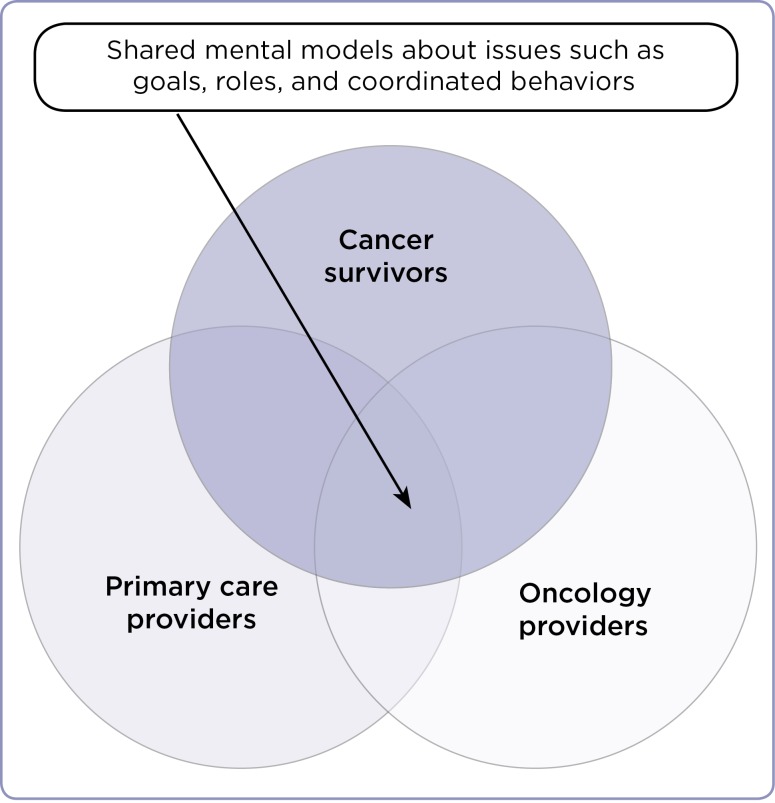
Mental models about survivorship.

The concept of shared mental models is particularly important in the health-care setting, when team members are frequently distributed across time and space (McComb & Hebdon, 2013; McComb et al., 2012). Therefore, we focus specifically on team members’ mental model content about provider roles, team goals, and coordinated behaviors, as this may provide insight regarding efficient and effective patient-centered survivorship care (see Figure 1). The following question was used to guide the literature search and data analysis: How do oncologists, primary care providers, and cancer survivors perceive the roles of oncologists and primary care providers in cancer survivorship care responsibilities?

## STUDY PURPOSE

The Institute of Medicine report discussed possible models of survivorship care but did not prescribe care provider roles or one model as best suited to this care area (Hewitt et al., 2005). In this review, we focus specifically on the question regarding maximizing the roles of providers in survivorship care. Quantitative and qualitative studies have evaluated care provider roles in the context of survivorship care, but no review has been completed.

Therefore, the purpose of this review is to analyze the convergent and divergent views of provider roles in survivorship care from the perspective of cancer survivors, primary care providers, and oncology providers. We employ the perspective that oncology providers, primary care providers, and cancer survivors are team members in cancer survivorship care (McComb and Hebdon, 2013). We also focus on the literature published from 2005 to the present to address the impact of the Institute of Medicine report on the perspectives of the key stakeholders.

## METHODOLOGY

**Search Strategy and Data Sources**

Systematic literature searches were performed using PubMed, CINAHL, and the Cochrane Database. Ancestry searches were also performed from the reference lists of retrieved articles. The search terms for PubMed were "primary care and cancer survivor follow-up or oncologist and cancer survivor follow-up." The search terms for the Cochrane Database were "oncologist and cancer survivor follow-up" and "primary care and cancer survivor follow-up." The search terms for CINAHL were "oncologist and cancer survivor follow-up" and "primary care and cancer survivor follow-up."

**Inclusion/Exclusion Criteria**

Article titles, abstracts, and text were reviewed to determine eligibility. An article was excluded if it was published before 2005, if the study was conducted outside the United States, if it was not primary research, or if it was not published in a peer-reviewed scientific journal. Articles were included based on the following criteria: discussed cancer survivor follow-up or survivorship care; addressed general survivorship care rather than a specific survivorship treatment issue; included oncology patients, oncologists, and/or primary care providers as participants; and addressed perceptions, views, experiences, and/or preferences of oncology patients, oncologists, and/or primary care providers regarding the roles of oncologists and primary care providers in survivorship care.

## RESULTS

**Study Selection and Characteristics**

**Figure 2:**
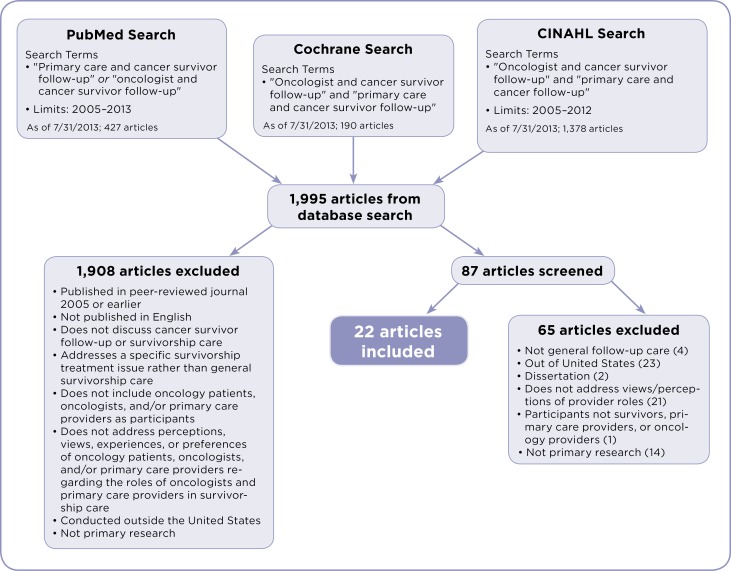
Details of search strategy.

A preliminary search resulted in 105 articles requiring final screening. These articles were reviewed by two authors, who had a 96% agreement regarding inclusion/exclusion. For the 4% not agreed upon, all three authors discussed the articles to ascertain their appropriateness for inclusion. Given the time lapse between the preliminary search and manuscript preparation, a second search, described in Figure 2, was completed using the same search criteria as the preliminary search. This database search resulted in 1,995 articles, which included all of the articles identified in the preliminary search. After duplicates, articles that met the exclusion criteria, and articles that did not meet inclusion criteria were removed, 87 articles were screened by one author. The decision to have one author screen the articles was justified by the high agreement obtained during the preliminary search.

For the final round of screening, titles, abstracts, and article text were reviewed to determine eligibility. Sixty-five articles were removed due to lack of compliance with the review parameters listed here. At the final stage of assessment, a total of 22 articles were included in the review. All authors were in agreement regarding the final set of articles and the final themes.

**Data Extraction and Synthesis**

Table 1 describes the articles, methodology, and participant type. Table 2 delineates the organization of primary and secondary themes based on the findings reported in 13 quantitative and 9 qualitative studies included in the analysis. The description of results includes both qualitative and quantitative data.

**Table 1:**
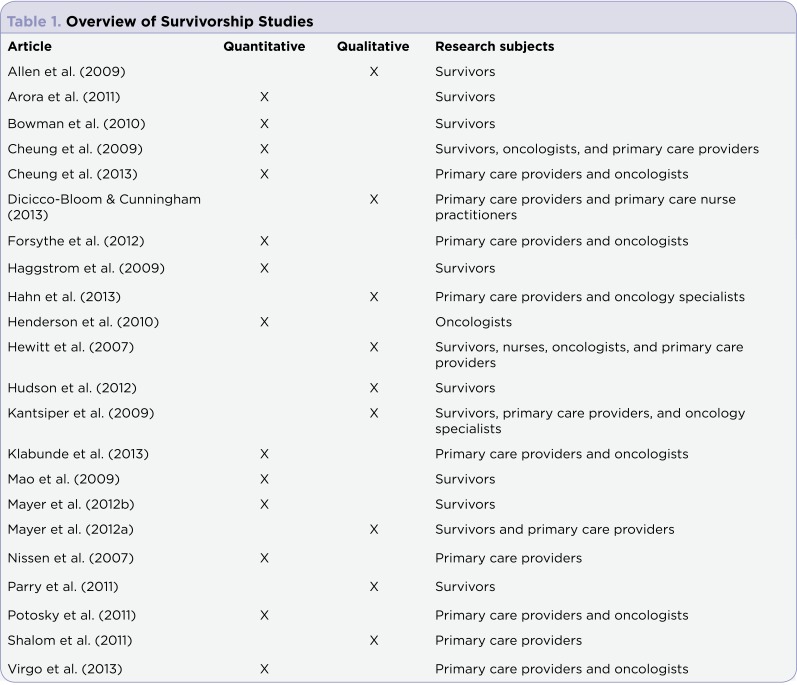
Overview of Survivorship Studies

**Needed Care**

Eight articles discussed care that is needed as cancer survivors transition from active treatment to follow-up care. The secondary themes for needed care included the psychological needs of cancer survivors, the need for follow-up and support, the use of a survivorship care plan, and ongoing health concerns of cancer survivors (see Table 2). Four articles noted emotional distress, fear, and cancer-related worry from the perspective of cancer survivors (Allen, Savadatti, & Levy, 2009; Bowman, Rose, Deimling, Kypriotakis, & O’Toole, 2010; Hewitt, Bamundo, Day, & Harvey, 2007; Parry, Morningstar, Kendall, & Coleman, 2011), with Hewitt et al. (2007) also noting that primary care providers recognize psychological stress in their cancer patients. Dicicco-Bloom & Cunningham (2013) reported that both primary care providers and oncology providers discussed the importance of psychological support for care continuity.

**Table 2:**
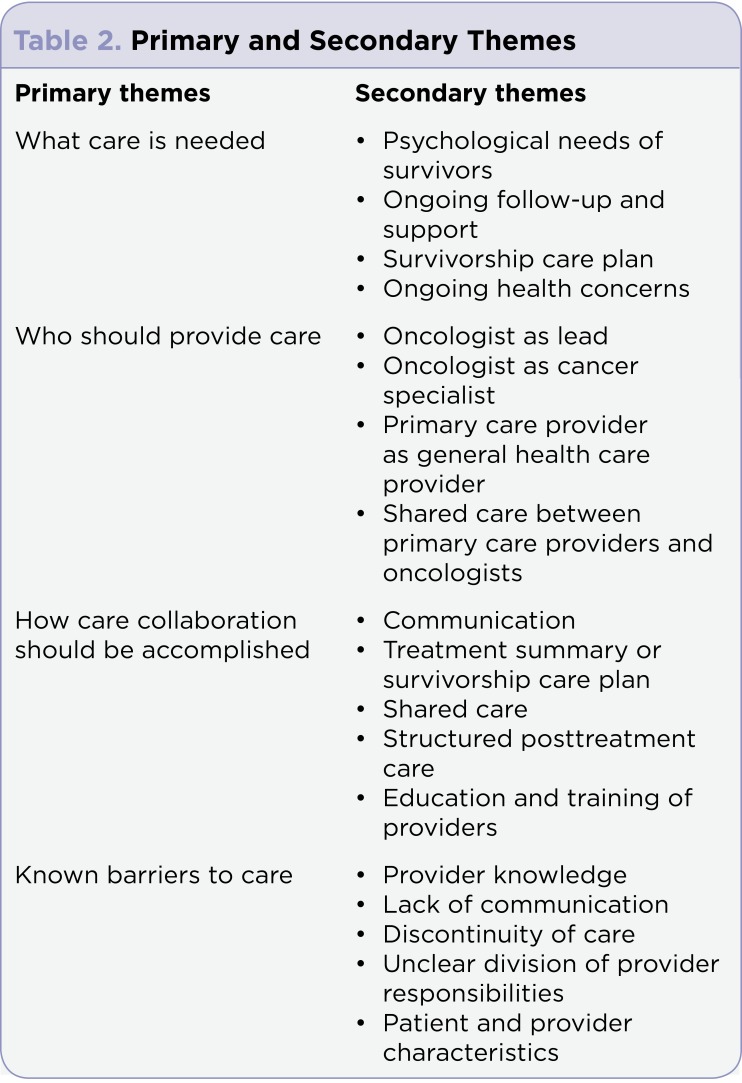
Primary and Secondary Themes

Allen et al. (2009), Dicicco-Bloom & Cunningham (2013), Hewitt et al. (2007), Kantsiper et al. (2009), and Parry et al. (2011) noted the importance of ongoing support, follow-up, and surveillance from the perspective of survivors, oncology providers, and primary care providers. According to Bowman and colleagues (2010), 29% of survivors reported cancer affecting their current health, and Kantsiper et al. (2009) discussed breast cancer survivors’ concerns regarding late effects that were not being addressed, such as weight gain, hot flashes, and sexual dysfunction.

Three articles discussed the need for a survivorship care or treatment plan from the cancer survivor perspective (Hahn et al., 2013; Hewitt et al., 2007; Parry et al., 2011), but Mayer, Gerstel, Leak, and Smith (2012a) noted that survivors felt the survivorship care plan might be too little, too late. Survivorship care plans were not described by either oncologists or primary care providers as being needed in any articles, but care coordination was noted by oncologists and primary care providers, according to Hahn et al. (2013), with subthemes of shared care, coordination across the institution, and care coordination within oncology. Finally, Parry et al. (2011) reported that survivors should be as prepared for survivorship as they were for treatment.

**Survivorship Care Providers**

Twelve articles discussed who should provide survivorship care from the perspective of cancer survivors, oncologists, and primary care providers. The secondary themes among the articles emphasized the oncologist having the primary cancer survivorship role, oncologists as the specialist, primary care providers as skilled in general health care, and shared care between oncologists and primary care providers (see Table 2). Among the articles, the consensus between patients and oncology providers is that oncologists should have the primary role, whereas primary care providers were divided regarding their role as lead physician in follow-up care, uninvolved, or involved in a shared-care approach. According to Cheung, Neville, Cameron, Cook, and Earle (2009); Haggstrom, Arora, Helft, Clayman, and Oakley-Girvani (2009); Hudson et al. (2012); Kantsiper et al. (2009); Mao et al. (2009); and Mayer et al. (2012b), survivors accessed or endorsed their cancer specialist for follow-up care related to their cancer. Oncologists also viewed themselves as the specialist and principal provider in cancer follow-up care (Cheung et al. [2009], Cheung et al. [2013], Kantsiper et al. [2009], Klabunde et al. [2013], and Potosky et al. [2011]).

Conversely, primary care providers were divided in their views as being the sole provider for follow-up care. According to Cheung et al. (2009) and Potosky et al. (2011), only 10% of primary care providers thought they should have full responsibility for cancer recurrence follow-up or preferred a primary care provider–led model, respectively. Primary care providers did not describe themselves as having a central role in survivorship care in the research by Kantsiper et al. (2009) as well.

Yet, in the research by Nissen et al. (2007), 52% of primary care providers reported being confident or very confident in performing surveillance for cancer recurrence in cancer survivors. Primary care providers also reported a preference for shared care or current practice of shared care, according to Cheung et al. (2013), Klabunde et al. (2013), and Potosky et al. (2011). Finally, survivors, oncologists, and primary care providers acknowledged the primary care provider role in general health care and/or the oncologist’s specialty role in the studies performed by Cheung et al. (2009), Forsythe et al. (2012), Haggstrom et al. (2009), Hudson et al. (2012), Kantsiper et al. (2009), Mao et al. (2009), and Mayer et al. (2012b).

**Care Collaboration**

Fifteen articles addressed how care collaboration should be accomplished between primary care providers and oncologists. Major themes throughout the articles included communication, use of a treatment summary or survivorship care plan, shared care, structured posttreatment care, and education and training of providers (see Table 2). In the research by Bowman et al. (2010), Kantsiper et al. (2009), Mao et al. (2009), and Parry et al. (2011), survivors reported improved care with communication or the need for communication throughout survivors and providers to improve care. According to Dicicco-Bloom & Cunningham (2013), Kantsiper et al. (2009), and Nissen et al. (2007), primary care providers discussed the importance of feedback, communication, and guidance from oncologists. Oncologists echoed the value of communication in the research by Kantsiper et al. (2009).

Oncologists, cancer survivors, and primary care providers discussed the impact or value of a treatment summary, written information, or a survivorship care plan (Dicicco-Bloom & Cunningham, 2013; Hahn et al., 2013; Hewitt et al., 2007; Kantsiper et al., 2009; Klabunde et al., 2013; Mayer et al., 2012a; Nissen et al., 2007; Parry et al., 2011; Shalom, Hahn, Casillas, & Ganz, 2011). Oncologists, survivors, and primary care providers thought that a communication tool such as a survivorship care plan would improve care (Dicicco-Bloom & Cunningham, 2013; Kantsiper et al., 2009; Shalom et al., 2011). Survivors and primary care providers both described the treatment summary or survivorship care plan as a way to guide follow-up care and provider responsibilities (Dicicco-Bloom & Cunningham, 2013; Mayer et al., 2012a; Nissen et al., 2007; Parry et al., 2011; Shalom et al., 2011).

Shared care was discussed or reported by primary care providers and/or oncologists in the research by Cheung et al. (2013), Hahn et al. (2013), Kantsiper et al. (2009), Klabunde et al. (2013), and Shalom et al. (2011). According to Klabunde et al. (2013), oncologists who preferred shared care were more likely to co-manage vs. other care models. In the study by Hahn et al. (2013), both primary care providers and oncologists reported shared care as a positive method of posttreatment care. Primary care providers demonstrated a willingness to lead, comanage, and have oncologists as active participants in the care of cancer survivors (Cheung et al., 2013; Kantsiper et al., 2009; Shalom et al., 2011).

Hudson et al. (2012) and Mao et al. (2009) discussed survivors’ views of shared care between primary care providers and oncologists. Survivors who perceived care cohesion for primary care providers and oncologists had higher ratings of perceived care delivery than those who did not report cohesive care between providers (Mao et al., 2009). According to Hudson et al. (2012), survivors described shared care as the only context in which a primary care provider should be involved in follow-up care.

Structured posttreatment care through a structured care transfer, survivorship clinics, a survivorship-specific clinician, cancer-specific primary care clinics, or multidisciplinary clinics were recommended by survivors, oncologists, and primary care providers in four articles (Hahn et al., 2013; Mao et al., 2009; Nissen et al., 2007; Potosky et al., 2011). Primary care providers reported the need for a more formal transfer of care in the article by Nissen et al. (2007). Breast cancer survivors expressed the desire for a designated primary care clinic for their needs (Mao et al., 2009). According to Hahn et al. (2013) and Potosky et al. (2011), primary care providers and oncologists supported specialized survivorship clinics and a specialized survivorship clinician. Oncologists supported both physician-led and nurse- or physician assistant–led clinics, whereas primary care providers preferred physician-led clinics (Potosky et al., 2011).

Provider training was reported or recommended by primary care providers, oncologists, and survivors as a factor in the care of cancer survivors in the articles by Henderson, Hlubocky, Wroblewski, Diller, and Daugherty (2010); Kantsiper et al. (2009); Klabunde et al. (2013); and Mao et al. (2009). In fact, primary care providers were more willing to be responsible for cancer follow-up care with ongoing training (Kantsiper et al., 2009) and more likely to lead or comanage care with training in late and long-term effects of cancer (Klabunde et al., 2013).

Survivors considered teaching primary care providers about issues for breast cancer survivors to be important or very important (Mao et al., 2009). Oncologists also demonstrated the need for training in the article by Henderson et al. (2010). Pediatric oncologists had better knowledge scores, with reports of familiarity with long-term follow-up guidelines and the receipt of training in the care of childhood cancer survivors.

**Known Barriers to Care**

Seventeen articles discussed the known or reported barriers to survivorship care provision. Major themes among the articles included provider knowledge, lack of communication, discontinuity of care, lack of understanding or consensus among providers and patients regarding provider roles, and patient characteristics (see Table 2). Provider knowledge of the patient and of cancer survivorship issues was a barrier reported by survivors, primary care providers, and oncologists in the studies by Arora, Reeve, Hays, Clauser, and Oakley-Girvan (2011); Cheung et al. (2013); Hahn et al. (2013); Henderson et al. (2010); Hudson et al. (2012); Nissen et al. (2007); Parry et al. (2011); Potosky et al. (2011); and Virgo, Lerro, Klabunde, Earle, and Ganz (2013).

Survivors noted that physicians had less-than-excellent knowledge of them individually, as well as limited knowledge about the effects of their cancer and its treatment on their quality of life (Arora et al., 2011). In multiple articles, lack of knowledge and preparation for cancer survivor follow-up needs was noted for both primary care providers and oncologists (Hahn et al., 2013; Henderson et al., 2010; Hudson et al., 2012; Parry et al., 2011; Potosky et al., 2011; Virgo et al., 2013).

Communication was a barrier reported by survivors and primary care providers in only six articles (Arora et al., 2011; Dicicco-Bloom & Cunningham, 2013; Hewitt et al., 2007; Kantsiper et al., 2009; Mao et al., 2009; Mayer et al., 2012a). Poor communication between providers and patients was noted by both Arora et al. (2011) and Hewitt et al. (2007), where survivors noted a lack of compassion, few questions about their health, and little to no discussion about health promotion and prevention. Survivors reported a perceived lack of communication between oncologists and primary care providers (Mao et al., 2009; Mayer et al., 2012a). Primary care providers reported poor or inconsistent communication from oncologists regarding mutual patients in the studies by Mayer et al. (2012a) and Kantsiper et al. (2009). Similarly, poor or inconsistent communication was noted by Dicicco-Bloom & Cunningham (2013), with primary care providers admitting to lack of communication on their part as well.

Discontinuity of care was addressed by survivors in the studies by Dicicco-Bloom & Cunningham (2013), Kantsiper et al. (2009), Mao et al. (2009), Mayer et al. (2012a), and Parry et al. (2011). According to both Kantsiper et al. (2009) and Parry et al. (2011), survivors noted feelings of abandonment at the end of treatment, with discontinuity in care and lack of knowledge about how to access help. According to Haggstrom et al. (2009), 27% of survivors reported not being seen for follow-up care. Survivors also rated care cohesion between primary care providers and oncologists as poor or average (56%) in the study by Mao et al. (2009).

Primary care providers reported variable experiences with continuity of care in the study by Mayer et al. (2012a), and 8.4% and 48.7% described the transfer of care from oncology as poor or fair, respectively, in the article by Nissen et al. (2007). Primary care providers noted a lack of interaction with patients during active treatment and felt excluded from the decision-making process (Dicicco-Bloom & Cunningham, 2013).

An unclear division of provider responsibilities in survivorship care activities was described or reported by survivors, primary care providers, and oncologists in the research by Cheung et al. (2013), Forsythe et al. (2012), Hahn et al. (2013), Kantsiper et al. (2009), Mayer et al. (2012a), and Potosky et al. (2011). According to Cheung et al. (2013), Forsythe et al. (2012), and Potosky et al. (2011), primary care providers and oncologists had differing views regarding primary care providers’ knowledge and abilities to perform follow-up care activities, with oncologists reporting lower ratings for primary care provider knowledge and involvement than primary care providers. Providers and survivors reported uncertainty regarding follow-up responsibilities for providers in the studies by Hahn et al. (2013) and Mayer et al. (2012a). Survivors also reported a perception that primary care providers do not want to overstep boundaries in follow-up care activities (Kantsiper et al., 2009).

Patient- and provider-specific barriers were discussed in six articles (Hewitt et al., 2007; Hudson et al., 2012; Kantsiper et al., 2009; Potosky et al., 2011; Shalom et al., 2011; Virgo et al., 2013). According to Hudson et al. (2012), survivors believed that a primary care provider would not include their history of cancer in diagnostic or treatment decisions. Primary care provider–specific barriers for survivorship care provision included information access, time, legal and malpractice concerns, correspondence volume from outside physicians, patient insurance or inability to pay, and lack of confidence in survivorship care plans prepared by nurse practitioners (Hewitt et al., 2007; Kantsiper et al., 2009; Shalom et al., 2011; Virgo et al., 2013).

Oncologist-specific barriers for survivorship care included rarely discharging patients to primary care providers for follow-up care, no time-saving or monetary benefit to treatment summary preparation, patient insurance or ability to pay, patient noncompliance with care, and patients requesting more aggressive surveillance than recommended (Hewitt et al., 2007; Potosky et al., 2011; Virgo et al., 2013).

## DISCUSSION

Our evidence demonstrates that team members have shared mental models as well as divergent perspectives regarding many issues associated with providers’ roles in the provision of survivorship care. Four primary themes emerged from the literature regarding what care is needed, who should provide that care, how care collaboration should be accomplished, and the known barriers to care. Within each of the themes, however, secondary themes arose, where team members may not have shared mental models; however, if they did, the quality of cancer survivorship care may have been markedly improved, such as the need for psychosocial support and ongoing follow-up (i.e., what care is needed), shared views of specialty and generalist roles (i.e., who should provide care), measures to promote care continuity including the survivorship care plan (i.e., how care collaboration should be accomplished), and discontinuity of care and provider knowledge that would inform teamwork behaviors among these individuals (i.e., known barriers to care). These issues and the previous research with which they align are highlighted in this discussion.

The literature is clear that ongoing support and follow-up are the elements of care needed by cancer survivors. Multiple articles noted feelings of abandonment by patients at the transition to follow-up care, uncertainty navigating survivorship care, and ongoing psychological and physical needs from the perspective of survivors (Allen et al., 2009; Bowman et al., 2010; Dicicco-Bloom & Cunningham, 2013, Hewitt et al., 2007; Kantsiper et al., 2009; Parry et al., 2011). These findings are not necessarily restricted to these studies or to the United States (Jefford et al., 2008). Cancer survivors have less contact with health-care providers when they are in follow-up care, leaving them to cope with the cancer experience without the support they had during treatment (National Coalition for Cancer Survivorship and Institute of Medicine, 2007). Survivors tellingly emphasized the need for the same level of support and education in follow-up care as they received during treatment (Kantsiper et al., 2009).

Specialist vs. generalist care was a pronounced secondary theme under the primary theme of who should provide care. Oncology providers and primary care providers have different, but equally important, roles in the care of cancer survivors. The primary care provider as the lead for wellness and psychosocial care (Cheung et al., 2009; Forsythe et al., 2012; Haggstrom et al., 2009; Mao et al., 2009; Mayer et al., 2012b) is consistent with the roles of primary care as identified by the American Academy of Family Physicians (AAFP): health promotion, disease prevention, health maintenance, and coordination of health care services (AAFP, 2013). The oncologist filling the primary role for cancer-focused care and less of a role in wellness and supportive care (Cheung et al., 2009; Forsythe et al., 2012: Haggstrom et al., 2009; Henderson et al., 2010; Klabunde et al., 2013; Mayer et al., 2012b; Potosky et al., 2011) best utilizes the oncology provider’s expertise (McCabe et al., 2013).

Facilitating care collaboration may not be easy, but treatment summaries or survivorship care plans were noted repeatedly. By employing these tools, team members may more effectively communicate, ensure care continuity, delineate provider roles, and support primary care providers’ decision-making (Dicicco-Bloom & Cunningham, 2013; Hahn et al., 2013; Hewitt et al., 2007; Kantsiper et al., 2009; Klabunde et al., 2013; Mayer et al., 2012a; Nissen et al., 2007; Parry et al., 2011; Shalom et al., 2011).

Consistent, concrete evidence to support the use of survivorship care plans in practice has yet to occur, although there is firm organizational support for their use as described previously (American College of Surgeons, 2012; Hewitt et al., 2005; McCabe et al., 2013). Oncology providers addressed time and financial barriers as prohibitive to their creating treatment summaries (Hewitt et al., 2007). Ideally, integrating survivorship care plans into electronic health systems would decrease the burden on oncology providers responsible for crafting the care plans (McCabe et al., 2013).

In addition to the aforementioned barriers associated with developing a meaningful survivorship care plan, other barriers that may inhibit the effective delivery of care to survivors were identified. In particular, provider knowledge for both oncologists and primary care providers was seen as a barrier to effective cancer survivorship care, and provider training was discussed as a positive method of promoting patient care (Arora et al., 2011; Hahn et al., 2013; Henderson et al., 2010; Hudson et al., 2012; Kantsiper et al., 2009; Klabunde et al., 2013; Nissen et al., 2007; Parry et al., 2011; Potosky et al., 2011; Virgo et al., 2013).

These findings highlight the need for ongoing training for all providers caring for cancer survivors. Some organizations, such as MD Anderson Cancer Center (MDACC), provide online continuing education opportunities for providers (MDACC, 2014). Survivors noted the need for more information on follow-up care (Kantsiper et al., 2009; Parry et al, 2011), which has also been noted in other research findings. Wheelock et al. (2013) reported significantly higher patient utilization of follow-up appointments with attendance at their survivorship group education.

## STUDY LIMITATIONS AND FUTURE DIRECTIONS

Limitations in this review may affect the broad application of our findings. Both quantitative and qualitative studies were included, and as the outcome measures among the quantitative studies were not redundant across studies, no statistical analysis was performed. Although this lack of statistical support makes the findings less powerful, the themes identified are still relevant. In addition, as only US studies were included in this review, the conclusions might not be translatable to the health-care structures of other nations. However, the overarching themes of provider roles and supportive measures for these roles could be incorporated into any model of health care. Moreover, this review did not formally account for the nursing role (or for the possibility of advanced practitioners functioning in survivorship roles, an ideal position for many of them), which is essential for a comprehensive view of the roles of health-care team members.

Although this review provides some consensus from health-care team members regarding which provider is best suited to particular care activities, the cost-effectiveness of these roles has not been evaluated. This could be accomplished through future evaluation of care models that utilize the cancer expertise of the oncologist and the comprehensive wellness focus of the primary care provider in the context of accountable care organizations or patient-centered medical homes to formally structure the health-care team.

Researchers should develop the idea of team member roles in cancer survivorship care to elucidate facilitators of teamwork. Nurses and patients’ families as key team members would further the research on teamwork in cancer survivorship care, as these two groups are fundamental to the health-care team (McComb & Hebdon, 2013). Survivorship care plans and survivorship care training could be explored further as mechanisms to promote team behavior among providers and patients.

## CLINICAL PRACTICE IMPLICATIONS

Along with future directions for research, there are direct clinical practice implications that can be drawn from this review. First, advanced practitioners, along with other members of the health-care team, can provide a structured process to ease the transition for patients from active treatment to follow-up care. This could include survivorship education for patients before, during, and after treatment; dedicated survivorship care visits; and the use of survivorship care plans to communicate patient needs with the health-care team.

Survivorship care plans also promote care continuity so oncology and primary care providers have a clear understanding of the respective specialty and overall wellness provider roles in survivorship care. Advanced oncology and primary care providers can seek out educational opportunities for survivorship care provision so they are equipped to address surveillance and the late effects of cancer survivors. Quality survivorship care requires structured and intentional behavior on the part of advanced care providers in meeting patient needs.

## CONCLUSION

Cancer survivorship requires coordinated team behaviors and corresponding shared mental models about those behaviors, from primary care providers, oncology providers, and patients. As survival rates improve and cancer survivors increase in number, quality-driven, cost-effective survivorship care models that best utilize the skills of oncology and primary care providers are paramount. Measures such as survivorship care training and survivorship care plans can aid health-care team members in maximizing the supportive and all-encompassing role of the primary care provider and the cancer expertise of the oncologist in providing patient-centered care.
